# Medico-Chirurgical Society of Montreal

**Published:** 1888-01

**Authors:** 


### MEDICO-CHIRURGICAL SOCIETY OF MONTREAL.

STATED MEETING, OCTOBER 28, 1887.

President J. Perrigo, M. D., in the Chair.

DR. JOHNSON exhibited (1) a microscopic section of a nail showing parasitic onychia, which had been sent to him by Dr. Bell. The chains of trychophyton were seen in moderate numbers in the deeper layer of the nail and between the nail and its bed.

DR. BELL gave the following history of the case: Miss E., aged twenty, in scraping the back of her thumb nail about a year ago, cut through it about the middle. A light brown spot developed at this point and gradually extended to its free margin, and then began to grow backwards towards the matrix. It was painless. When seen the anterior two-thirds of the nail was dull and dry-looking, yellowish-brown in color, and raised from its bed at the free anterior margin to the extent of nearly half an inch. The tissue between the nail and its bed, at the margin, was quite dry and cancellated, resembling the cancellated structure of a dry bone. The nail was removed by slitting down the center and removing the two portions separately. This cancellated structure was separated from the nail-bed by a thin fibrous layer, beneath which the nail-bed was absorbed. Owing to its peculiar appearance the nail was macerated and sections cut through the diseased part. On examination, there showed in considerable quantities the mycelium and spores of the trichophyton, resembling the fungus as seen in tinea circinata rather than as usually seen in T. tonsurans. There was no history of tinea on this patient's skin, nor, as far as she knew, on other members of her family.

*Broncho-pneumonia.*—(2) A microscopic section through the lung of a sheep in a case of broncho-pneumonia, where great numbers of the embryos or strongylus filaria were found in the alveoli, which were filled with exudation, and there was severe bronchitis and peribronchitis of the smaller tubes. The adult forms were not found within the bronchi, having probably been coughed up. The embryos are not able to develop beyond this stage in the lung.

*Amputation of the thigh.*— DR. BELL exhibited a patient whose thigh had been amputated for periosteal sarcoma. The specimen had been exhibited at a previous meeting. The patient was eighteen years of age, and at the time of the operation had been in a very low state of health. His temperature ranged from 100°F. to 103½°F., his pulse from 120 to 140 per minute, and he was greatly emaciated. Amputation was performed by the circular method, about two inches below the base of the trochanter major, on the 3d of October, and from that time his condition improved rapidly. His temperature remained steadily at 98½°, and he rapidly gained flesh. The dressing was changed once only, on the eighth day, and removed on the twenty-fourth day after the operation, when the stump was perfectly healed.

*Osteotomy for bow-legs.*— A child, three and one-half years of age was shown to the Society, on whom Dr. Bell had performed double osteotomy. The condition was the result of rickets, from which the child had perfectly recovered. The operation had been performed by MacEwen's method, and had resulted very favorably. Photographs were shown of the child's legs before the operation.

*Discussion.*— DR. RODDICK referred to the good results obtained by Dr. Bell in using bone drains. His experience with this mode of draining was not so favorable, as he found that the bone drains were too rapidly absorbed. While he congratulated Dr. Bell on the excellent results obtained in his operation for bow-legs, yet he could not agree with the necessity for the operation. Dr. MacEwen, who introduced the operation, does not recommend its application in patients under nine years. He (Dr. Roddick) had obtained quite as good results from the use of mechanical contrivances in children even older than the patient. He thought that in most cases subcutaneous fracture is to be preferred to osteotomy, as it is a less serious operation, and offers less risk. While opposed to operations in most of these cases of deformity, he thought it was more frequently demanded in knock-knees than in bow-leg, as the former requires much longer and more painful treatment.

Dr. Shepherd said that in one of the few times when he had used bone drains, he found the patient's temperature had risen and the drain plugged with a clot. He always prefers using rubber drains, which he cuts down to three-quarters of an inch at the end of twenty-fours. In Germany the "single-dressing" mania often results disastrously to the patient. In German hospitals he was frequently shown single-dressing cases where the temperature chart indicated an unhealthy condition of the wound. He had seen Dr. Bell's patient before the operation, and he could heartily congratulate him on the success. With regard to the osteotomy case, he referred to the erroneous but common opinion that all cases of bow-legs result from rickets. The peculiarity is often hereditary, and is quite normal in many of the anthropoid apes.

Dr. Armstrong referred to Dr. Levis' system of drainage. He uses solid rubber strings placed side by side, instead of tubes, thus obviating the danger of plugging.

Dr. Gurd said he had seen very good results from treatment of bow-legs by improving the general health. He had great faith in the efficacy of good hygienic surroundings and the use of tonics in such cases. Instruments have proved unsatisfactory.

Dr. Bell, in reply, stated that the drains used were made from chicken bones, by the method recommended by Dr. MacEwen, of Glasgow. These could be obtained as hard or as soft as desired. In the case of osteotomy, the curve in the child's legs was greatest just above the malleolus, so it could not be treated by subcutaneous fracture.

*Notes on acetanilide.*—Dr. McConnell first briefly stated what was known about acetanilide or antifebrin up to the present time. It is procured from aniline acetate, is a white powder resembling santonin, insoluble in water, but soluble in alcohol. It is neither alkaline nor acid, and resists the majority of re-agents. It belongs to the order *Phenylacetamides*, quite different from the orders containing the majority of antipyretics, viz., the Phenols and Chinolins. Actions claimed for it are that it rapidly reduces the temperature in febrile states, without producing any untoward effects; that it is also hypnotic and analgesic, being especially useful in relieving pain linked with nerve alterations. In poisonous doses it will destroy oxyhæmoglobin, changing it into methæmoglobin. It is inexpensive, being only 10 francs per one kilogramme in France. He had used it in about twenty cases, of sixteen of which he had records — nine were cases of typhoid fever — in all of which the temperature was promptly reduced.

*Discussion.*— Dr. Proudfoot had used acetanilide in painful affections of the eye, such as iritis and glaucomata, in doses of ten to fifteen grains. He found it reduced the temperature and relieved the pain almost instantly. If the pain was not relieved in one hour, he usually repeated the dose.

Dr. Stewart said he had very little experience in the use of the drug. He had, however, administered it in five-grain doses to relieve the lightning pains of locometer ataxia, and found it very efficient. He regarded it as dangerous to give powerful drugs in fever cases to reduce temperature, as these act on the oxyhæmoglobin, thus reducing the patient's powers of resistance.

Dr. Reed stated that from Dr. Charcot's recommendation he had used it, but had not been able to relieve pain. He had found it to reduce the temperature for a time, though not sufficiently to encourage him to continue its use.

Dr. Perrigo said that the drug failed entirely in a case of malaria, in which he had tried it.

Dr. Roddick congratulated Dr. McConnell on his finding something to relieve the distressing headache of typhoid. He had given it in a case of erysipelas, but it had no effect on the temperature.

Dr. Blackader had also administered the drug in erysipelas with very little effect. The German authorities state that it is without effect in scarlet fever and erysipelas. He thought, however, that the anodyne properties of the drug would keep it in the pharmacopœia.

In reply to remarks of Dr. Stewart, that its action on oxyhæmoglobin was an objection to its use, Dr. McConnell said that this only occurs to any appreciable extent when over-doses are taken. The antipyretic action is almost altogether exerted through the nervous system, and chiefly the vaso-motor. The want of effect in cases referred to by Drs. Reed and Perrigo was probably owing to its having been administered in too small doses.

